# Association Between Sleep Quality and Depressive Symptoms in Adults Aged 80 Years and Above Living in Rural Türkiye: A Community-Based Cross-Sectional Study

**DOI:** 10.7759/cureus.96051

**Published:** 2025-11-03

**Authors:** Denise Oyku Inanc, Hatice S Ercin

**Affiliations:** 1 General Practice, Tuzlukcu State Hospital, Konya, TUR; 2 Family Medicine, Tuzlukcu State Hospital, Konya, TUR

**Keywords:** age and depression, aging population, geriatric depression scale, geriatric health, pittsburgh sleep quality index (psqi), rural türkiye, sleep quality

## Abstract

Background: Sleep and mood disorders are common health problems in older adults and can have a marked impact on overall well-being. Poor sleep quality has been linked to cognitive decline, frailty, and depressive symptoms, all of which can reduce independence and quality of life. Older adults in rural areas may face added challenges such as limited healthcare access, financial hardship, and social isolation. Examining how sleep quality and mood interact in this group, alongside their sociodemographic characteristics, is important for identifying ways to support healthy aging. This study investigated the relationship between sleep quality and depressive symptoms in adults aged 80 years and older living in rural Türkiye.

Methodology: This cross-sectional study was carried out between October 2024 and January 2025 in Tuzlukçu, a rural district of Konya, Türkiye. Data were collected from 205 participants aged 80 to 101 years using the Pittsburgh Sleep Quality Index (PSQI) and the 30-item Geriatric Depression Scale (GDS). Sociodemographic information was obtained through structured interviews. Descriptive statistics, chi-square tests, and Spearman’s correlation analyses were used to explore associations between sleep quality, depressive symptoms, and participant characteristics.

Results: Nearly half of the participants (97, 47.3%) reported poor sleep quality, and 39.6% showed signs of depression (61, 29.8% mild; 20, 9.8% severe). A positive correlation was found between PSQI and GDS total scores (*r* = 0.361, *P* < 0.001), indicating that poorer sleep quality was linked to higher levels of depressive symptoms.

Conclusions: Poor sleep quality was closely associated with depressive symptoms among adults aged 80 years and older in rural Türkiye. Regular assessment of sleep and mood in community-based elder care, along with education on sleep hygiene and social support initiatives, may help improve mental well-being in this population.

## Introduction

Population aging is considered one of the most significant social transformations of the twenty-first century. By 2050, the number of people aged 60 and over is expected to double, reaching 2.1 billion globally [1]. As populations age, addressing age-related health concerns, including sleep quality and mental well-being, becomes increasingly urgent across healthcare systems worldwide [2]. Türkiye is among the countries experiencing a rapid demographic shift. The population aged 65 and over increased by 20.7% between 2019 and 2024, rising from 7.55 million to 9.11 million. Their share of the total population grew from 9.1% to 10.6%, with 55.4% of the elderly being female. Projections estimate that the elderly population will constitute 13.5% of the total population by 2030, 27.0% by 2060, and 33.6% by 2100 [3]. With the growth of the elderly population, there is an increasing need for comprehensive health strategies that address their physical, mental, and social well-being. Understanding the demographic dynamics and related health concerns at both global and national levels is essential for developing sustainable public health policies.

Sleep is a fundamental biological need, especially later in life, when restorative rest becomes increasingly important for maintaining physical and mental health. Older adults are recommended to sleep seven to eight hours per night; however, many fail to meet this duration [4]. Epidemiological evidence suggests that short sleep duration and poor sleep quality are associated with premature mortality, a wide range of adverse health outcomes, reduced quality of life, and increased levels of unhappiness [5].

In older age, sleep disturbances are closely tied to mental and neurological conditions. More than 20% of people aged 60 years and above are affected by such disorders. Among these, dementia and depression are the most prevalent, impacting approximately 5% and 7% of older adults, respectively. Moreover, disparities in health outcomes among older adults are often influenced by their place of residence, with rural populations facing unique challenges compared to their urban counterparts. Studies have consistently shown that rural populations are more likely to have lower income and education levels, live in environments with less developed physical and social infrastructure, and face barriers in accessing healthcare services. These circumstances may contribute to increased susceptibility to psychological distress [6,7]. For example, in China, the prevalence of depression in rural areas was found to be 1.5 times higher than in urban areas [8]. One study reported a psychological distress rate of 33.4% in a Chinese rural sample, reflecting the scale of mental health concerns in such settings [9].

Despite growing awareness of these issues, most research has focused on urban populations, leaving a gap in understanding the relationship between sleep quality and psychological well-being among rural older adults [10,11]. Understanding these dynamics in relation to sociodemographic factors may offer valuable insights into the unique challenges faced by older adults living in rural areas and help guide the development of tailored mental health interventions and support strategies. In this context, the present study investigates the relationship between sleep quality, depressive symptoms, and sociodemographic variables among individuals aged 80 and above residing in rural Türkiye. Cognitive impairment is a particularly important consideration when assessing this relationship, as it may reduce the accuracy of participants’ self-reported sleep and mood data. To improve data reliability, the study includes only participants without diagnosed cognitive impairment.

## Materials and methods

Study design and setting

This cross-sectional, descriptive study was conducted between October 2024 and January 2025 in Tuzlukçu, a rural district in Konya, Türkiye. The study focused on individuals aged 80 years and older who were registered with the YAŞAM Unit of Tuzlukçu Şehit Halim Ünal State Hospital. Ethical approval was obtained from the Non-Interventional Clinical Research Ethics Committee of Necmettin Erbakan University (2024/5216, application date: September 18, 2024).

Study population and participants

Tuzlukçu, with a population of 6.546 as of 2023, has a notably high proportion (21.93%) of residents aged 65 years and above, making it a relevant setting for aging-related research in rural Türkiye [3]. Participants were recruited exclusively using a simple random sampling method from individuals who were enrolled in the YAŞAM (Sağlıklı Yaş Alma Merkezleri) Program, a national initiative launched under Circular 2023/5 by the Turkish Ministry of Health, which specifically serves individuals aged 80 and older. The YAŞAM program aims to improve healthcare access for the elderly population through regular home visits, needs assessments, continuity of care, and support services, including remote consultation and transportation when needed.

Data collection was carried out during routine home visits conducted by trained healthcare professionals affiliated with the hospital's YAŞAM Unit. Although the research was not formally embedded within the YAŞAM program, the existing infrastructure facilitated access to a vulnerable and often underrepresented population in a community-based setting.

The target population consisted of 264 individuals aged ≥80 years. Based on a 95% confidence level and a 5% margin of error, the minimum sample size required was calculated as 215. Ultimately, 205 participants were included in the study (mean age: 84.88 ± 4.13 years; range: 80-101 years). Ten individuals were excluded due to hospitalization and mortality during the study period.

Inclusion criteria were being 80 years of age or older, residing in Tuzlukçu district, able to understand and provide verbal and written informed consent, either personally or via a legal guardian. Exclusion criteria included the presence of cognitive or psychiatric impairments that would prevent reliable participation. Cognitive impairments were assessed using the Mini-Mental State Examination (MMSE), with a score of less than 9 considered indicative of severe cognitive impairment; such individuals were excluded from the study [12].

Data collection instruments

Data were collected through structured, face-to-face interviews conducted during home visits by trained healthcare staff.

Sleep Quality Assessment

Sleep quality was evaluated using the Turkish validated version of the Pittsburgh Sleep Quality Index (PSQI), developed by Buysse et al. [13] and adapted by Ağargün et al. [14]. The PSQI is a self-reported questionnaire that assesses sleep quality over the past month. It includes 19 items grouped into seven components: subjective sleep quality, sleep latency, sleep duration, habitual sleep efficiency, sleep disturbances, use of sleeping medication, and daytime dysfunction. Each component is scored from 0 (no difficulty) to 3 (severe difficulty), resulting in a global score ranging from 0 to 21. A total score >5 indicates poor sleep quality. The PSQI is widely used in both clinical and research settings due to its practicality and strong psychometric validity.

Assessment of Depression

Depressive symptoms were measured using the 30-item Geriatric Depression Scale (GDS), originally developed by Yesavage et al. [15] and validated in Turkish by Ertan and Eker [16]. The GDS is a self-report tool designed specifically for older adults. Each item is answered with “yes” or “no.” Twenty of the items are scored as 1 for “yes” and 0 for “no,” while the remaining 10 are reverse-scored. The total score ranges from 0 to 30, with higher scores indicating more severe depressive symptoms. Scores are categorized as follows: 0-9 = no depression, 10-19 = mild depression, and 20-30 = severe depression.

Demographic Data Collection

A researcher-developed demographic questionnaire was used to collect baseline sociodemographic and health-related information. The form included items on age, gender, educational attainment (from illiterate to postgraduate), marital status, household composition (e.g., living alone, with spouse, children, or others), and self-reported income level (low, moderate, or high). Participants were also asked whether they had children, any chronic physical or psychological conditions, and whether they were currently using any medications. Lifestyle and health risk factors were assessed through questions on smoking status (current, former, or never) and history of falls resulting in fractures within the past five years.

Statistical analysis

Data were analyzed using IBM SPSS Statistics version 26.0 (IBM Corp., Armonk, NY). Descriptive statistics, including mean, standard deviation (SD), frequency, and percentage, were calculated for relevant variables. The normality of continuous variables was assessed by examining skewness and kurtosis values, with an acceptable range of −1.5 to +1.5. Variables that did not meet normality assumptions were analyzed using non-parametric tests. Spearman’s correlation analysis was conducted to evaluate the relationships between PSQI and GDS scores, including their respective subcomponents. Associations between sleep quality or depression levels and sociodemographic variables were examined using chi-square tests. A *P *< 0.05 was considered statistically significant.

## Results

Sociodemographic characteristics

The study sample consisted of a total of 205 participants, including 123 (60.0%) female and 82 (40.0%) male participants. Regarding educational status, 199 (96.6%) participants had a primary school education or below, while only 6 (3.0%) had completed middle school or higher. In terms of living arrangements, 34 (16.6%) participants lived alone, 90 (43.9%) lived with a spouse, 78 (38.0%) lived with their children, and 3 (1.5%) lived with other family members. With respect to income levels, 114 (55.6%) participants were in the low-income group, whereas 91 (44.4%) were in the middle- or high-income groups. Marital status distribution indicated that 98 (47.8%) participants were married, 8 (3.9%) were single, and 99 (48.3%) were widowed. A total of 148 (72.2%) participants had at least one chronic illness, and 142 (69.3%) reported regular medication use. Additionally, 175 (85.4%) participants were non-smokers, and 192 (93.7%) reported no history of falls within the past month. Participants’ ages ranged from 80 to 101 years, with a mean age of 84.88 ± 4.13 years (Table 1).

**Table 1:** Sociodemographic characteristics of the participants.

Variable	Category	n	%/Mean ± SD
Gender	Female	123	60.0
	Male	82	40.0
Education level	Illiterate	79	38.5
	Literate	29	14.1
	Primary School	91	44.4
	Secondary and higher	6	3
Living situation	Alone	34	16.6
	With family members	171	83.4
Age	Range	80-101	84.88 ± 4.13
Income level	Low	114	55.6
	Stabile	91	44.4
Marital status	Married	98	47.8
	Single	8	3.9
	Partner loss	99	48.3
Chronic disease	Yes	148	72.2
	No	57	27.8
Medication use	Yes	142	69.3
	No	63	30.7
Smoking status	Current smoker	13	6.3
	Non-smoker	175	85.4
	Former smoker	17	8.3
History of falls (past month)	Yes	13	6.3
	No	192	93.7

According to Table 2, based on the distribution of GDS scores, 124 participants (60.5%) were classified as not having depression, 61 participants (29.8%) had mild depression, and 20 participants (9.8%) had severe depression. In terms of sleep quality as assessed by the PSQI, 108 participants (52.7%) were found to have good sleep quality, while 97 participants (47.3%) had poor sleep quality. Participants’ mean GDS score was 9.20, and the mean PSQI score was 4.93.

**Table 2:** Distribution of GDS and PSQI scores. PSQI, Pittsburgh Sleep Quality Index; GDS, Geriatric Depression Scale

Scale	Classification	Number of participants, *n*	%
Geriatric Depression Scale	No Depression	124	60.5
	Mild Depression	61	29.8
	Severe Depression	20	9.8
Pittsburgh Sleep Quality Index	Good Sleep Quality	108	52.7
	Poor Sleep Quality	97	47.3

Associations between PSQI, GDS, and sociodemographic variables

Associations between sleep quality or depression levels and sociodemographic variables were examined using chi-square tests.

GDS scores were significantly associated with gender (χ² = 6.719, *P* = 0.035). Although there were more female participants overall, a higher percentage of males were in the non-depressed group, indicating that males were relatively more likely to be non-depressed than females. A significant relationship was also observed between GDS scores and marital status, where single and married participants were more likely to be classified as non-depressed.

Statistically significant relationships between GDS scores and the sociodemographic variables of gender and marital status are presented in Table 3.

**Table 3:** Relationship between sociodemographic variables and GDS scores (with P-values). A *P*-value < 0.05 was considered statistically significant. Variables were examined using chi-square tests. GDS, Geriatric Depression Scale

Variable	Category	Number of participants, *n* (%)	GDS: No depression, *n* (%)	GDS: Mild, *n* (%)	GDS: Severe, *n* (%)	X^2^	*P *(GDS)
Gender	Female	123 (60%)	68 (55.3%)	38 (30.9%)	17 (13.8%)	6.719	0.035
	Male	82 (40%)	56 (68.3%)	23 (28%)	3 (3.7%)		
Education	Illiterate	79 (38.5%)	44 (55.7%)	25 (31.6%)	10 (12.7%)	5.079	0.886
	Literate	29 (14.1%)	17 (58.6%)	10 (34.5%)	2 (6.9%)		
	Primary	91 (44.4%)	58 (63.7%)	25 (27.5%)	8 (8.8%)		
	Secondary and above	6 (3%)	5 (83.3%)	1 (16.7%)	0		
Living situation	Alone	34 (16.6%)	20 (58.8%)	11 (32.4%)	3 (8.8%)	4.402	0.622
	with family members	171 (83.4%)	102 (59.6%)	50 (29.2%)	17 (9.9%)		
Income	Low	114 (55.6%)	63 (55.3%)	41 (36%)	10 (8.8%)	5.639	0.223
	Stabile	91 (44.4%)	61 (67%)	20 (22%)	10 (11%)		
Marital status	Married	98 (47.8%)	66 (67.3%)	25 (25.5%)	7 (7.1%)	5.714	0.017
	Single	8 (3.9%)	7 (87.5%)	1 (12.5%)	0		
	Partner Loss	99 (48.3%)	51 (51.5%)	35 (35.4%)	13 (13.1%)		
Chronic illness	Yes	148 (72.2%)	84 (56.8%)	48 (32.4%)	16 (10.8%)	3.113	0.211
	No	57 (27.8%)	40 (70.2%)	13 (22.8%)	4 (7%)		
Medication use	Yes	142 (69.3%)	83 (58.5%)	45 (31.7%)	14 (9.9%)	0.903	0.637
	No	63 (30.7%)	41 (65.1%)	16 (25.4%)	6 (9.5%)		
Smoking	Smoker	13 (6.3%)	10 (76.9%)	3 (23.1%)	0	5.437	0.245
	Non-smoker	175 (85.4%)	101 (57.7%)	54 (30.9%)	20 (11.4%)		
	Former smoker	17 (8.3%)	13 (76.5%)	4 (23.5%)	0		
Fall history	Yes	13 (6.3%)	6 (46.2%)	6 (46.2)	1 (7.7%)	1.786	0.409
	No	192 (93.7%)	118 (61.5%)	55 (28.6%)	19 (9.9%)		

There was a significant association between PSQI scores and income levels, with participants in the low-income group more likely to report good sleep quality. Similarly, a significant relationship was found between PSQI scores and history of falls in the past month, participants without a recent fall were more likely to have good sleep quality (Table 4). 

**Table 4:** Relationship between sociodemographic variables and PSQI scores (with P-values). A *P*-value < 0.05 was considered statistically significant (bold values). Variables were examined using chi-square tests. PSQI, Pittsburgh Sleep Quality Index

Variable	Category	Number of participants, *n* (%)	PSQI: Good, *n* (%)	PSQI: Poor, *n* (%)	*X*^2^	*P* (PSQI)
Gender	Female	123 (60%)	61 (49.6%)	62 (50.4%)	1.177	0.278
	Male	82 (40%)	47 (57.3%)	35 (42.7%)		
Education	Illiterate	79 (38.5%)	43 (54.4%)	36 (45.6%)	3.507	0.622
	Literate	29 (14.1%)	15 (51.7%)	14 (48.3%)		
	Primary	91 (44.4%)	47 (51.6%)	44 (48.4%)		
	Secondary and above	6 (3%)	3 (50%)	3 (50%)		
Living situation	Alone	34 (16.6%)	17 (50%)	17 (50%)	0.384	0.944
	With family members	171 (83.4%)	91 (53.2%)	80 (46.8%)		
Income	Low	114 (55.6%)	69 (60.5%)	45 (39.5%)	6.380	0.041
	Stabile	91 (44.4%)	39 (42.9%)	52 (57.1%)		
Marital status	Married	98 (47.8%)	50 (51%)	48 (49%)	1.176	0.555
	Single	8 (3.9%)	3 (37.5%)	5 (62.5%)		
	Partner loss	99 (48.3%)	55 (55.6%)	44 (44.4%)		
Chronic illness	Yes	148 (72.2%)	76 (51.4%)	72 (48.6%)	0.379	0.538
	No	57 (27.8%)	32 (56.1%)	25 (43.9%)		
Medication use	Yes	142 (69.3%)	71 (50%)	71 (50%)	1.334	0.248
	No	63 (30.7%)	37 (58.7%)	26 (41.3%)		
Smoking	Smoker	13 (6.3%)	8 (61.5%)	5 (38.5%)	0.442	0.802
	Non-smoker	175 (85.4%)	91 (52%)	84 (48%)		
	Former smoker	17 (8.3%)	9 (52.9%)	8 (47.1%)		
Fall history	Yes	13 (6.3%)	3 (23.1%)	10 (76.9%)	4.881	0.027
	No	192 (93.7%)	105 (54.7%)	87 (45.3%)		

Relationship between PSQI and GDS categories

Spearman’s correlation analysis revealed a significant positive correlation between GDS scores and total PSQI scores, indicating that poorer sleep quality was related to higher levels of depressive symptoms.

At the subscale level, GDS scores were significantly positively correlated with subjective sleep quality, sleep latency, habitual sleep efficiency, sleep disturbances and daytime dysfunction.

No significant correlations were found between GDS scores and Sleep Duration or Use of Sleep Medication (Table 5).

**Table 5:** Spearman’s correlation coefficients among GDS, PSQI total, and subcomponents. A *P*-value < 0.05 was considered statistically significant (bold values). PSQI, Pittsburgh Sleep Quality Index; GDS, Geriatric Depression Scale

PSQI component	*r* with GDS	P
PSQI Total Score	0.361	<0.001
Subjective Sleep Quality	0.364	<0.001
Sleep Latency	0.228	<0.001
Sleep Duration	0.132	0.051
Habitual Sleep Efficiency	0.229	<0.001
Sleep Disturbances	0.274	<0.001
Use of Sleep Medication	0.047	0.465
Daytime Dysfunction	0.240	<0.001

## Discussion

This study examined the relationship between perceived sleep quality and depression among elderly individuals aged 80 and above living in rural areas. Consistent with previous research, Spearman’s correlation analysis revealed a significant positive correlation between GDS scores and both PSQI total and several subcomponent scores, indicating that greater depressive symptomatology was associated with poorer sleep quality.

The observed association between depressive symptoms and poor sleep quality aligns with a growing body of research highlighting a bidirectional relationship between these variables [17]. While it is well-documented that depression can impair sleep initiation and maintenance, poor sleep quality has also been shown to precede and predict the onset of depressive episodes [18,19]. This bidirectional mechanism is particularly concerning in the geriatric population, where multimorbidity, functional decline, and reduced access to healthcare may accelerate the negative cycle between poor sleep and mood dysregulation [20]. In this study, GDS scores were significantly associated with subjective sleep quality, sleep latency, habitual sleep efficiency, and daytime dysfunction, reinforcing the multifaceted nature of this relationship.

Sleep disturbances in late life are critical indicators of broader health vulnerabilities. For example, research from Korea, China, and Brazil has demonstrated strong correlations between poor PSQI scores and frailty, reduced life satisfaction, and worsened chronic disease control [9,17,21]. In our study there was significant association between good sleep quality and absence of falls. Falls are a major cause of morbidity and loss of independence in the elderly. Poor sleep has been linked to impaired balance, delayed reaction times, and nighttime disorientation, all of which increase the risk of falls [22]. Furthermore, poor sleep quality in older adults has been associated with impaired glucose metabolism, accelerated cognitive decline, and increased mortality risk [23,24].

Consistent with previous research, the current study found that female participants were more likely to be depressed than male participants (GDS scores were significantly associated with gender, χ² = 6.719, p = 0.035), with a higher proportion of males being non-depressed relative to their group size, despite females being more numerous overall [25]. However, some studies support the opposite pattern. For example, a longitudinal cohort study using the Obvious Depression Scale observed that gender differences in depressive symptoms diminished by age 80, with men showing a late-life increase in depression not seen in women [26].

Depression was also significantly related to living alone, supporting evidence that social isolation is a potential risk factor for late-life depression [27,28]. Similarly, being married was associated with lower depression scores, which aligns with previous findings that marital relationships can buffer against emotional distress through companionship and care [29].

In contrast to much of the existing literature, participants with lower income were more likely to report better sleep quality [30]. This counterintuitive finding may reflect cultural differences in the perception of sleep, reduced awareness or reporting of sleep problems, or differing expectations regarding health and rest in older rural populations. It is also possible that lower-income individuals in this age group maintain more stable daily routines and encounter fewer external stressors. These findings highlight the importance of contextual and cultural considerations when interpreting self-reported sleep outcomes.

Sleep duration and the use of sleep medication were not significantly related to depressive symptoms in this population. This suggests that subjective perceptions of sleep, rather than objective sleep quantity or pharmacological interventions, may be more critical in shaping mood outcomes in the elderly. In particular, prolonged sleep latency and poor subjective evaluations of sleep emerged as notable contributors to depressive symptomatology.

Despite providing valuable insights, the study has several limitations. Its cross-sectional design limits causal interpretations. The sample was drawn from a single rural district, and all data were self-reported, which may introduce recall and reporting biases. Additionally, the low number of single participants may have contributed to the lack of significant findings related to marital status, and the unequal gender distribution (123 female, 82 male) may have affected the generalizability of the findings across genders. Nevertheless, the focus on individuals aged 80 years and older in rural areas, an understudied and highly vulnerable group in geriatric mental health research, represents an important strength of this study.

## Conclusions

Sleep in older adults is not only a marker of overall well-being but also a modifiable factor in preventive mental health care. The findings underscore the importance of routine screening for sleep and mood disturbances in elderly individuals, particularly in rural areas. Incorporating sleep hygiene education, psychosocial interventions, and community-based support into primary care may help mitigate the burden of depression in this population.

The context of this study adds an important perspective to geriatric sleep and mental health research. In rural regions of Turkey, healthcare access, psychiatric service availability, and health literacy may be limited, which can delay the detection and management of sleep and mood disorders. Moreover, older adults in rural settings often experience isolation, bereavement, and decreased social engagement - all of which may contribute to both depression and disrupted sleep. These findings highlight the need for culturally sensitive, community-based programs that address both domains simultaneously.

Future research should adopt longitudinal and interventional designs to clarify causal pathways and evaluate the effectiveness of targeted strategies aimed at improving both sleep and mental health outcomes.
